# Estimates of linkage disequilibrium and effective population sizes in Chinese Merino (Xinjiang type) sheep by genome-wide SNPs

**DOI:** 10.1007/s13258-017-0539-2

**Published:** 2017-04-17

**Authors:** Shudong Liu, Sangang He, Lei Chen, Wenrong Li, Jiang Di, Mingjun Liu

**Affiliations:** 1 College of Animal Science and Technology, Shihezi University, Shihezi, Xinjiang China; 2 Key Laboratory of Genetics Breeding and Reproduction of Grass feeding Livestock, MOA, Urumqi, Xinjiang China; 3 The Key Laboratory of Animal Biotechnology of Xinjiang, Xinjiang Academy of Animal Science, Urumqi, Xinjiang China; 4 Xinjiang Wool sheep and Cashmere Goat Key Breeding Lab, Institute of Animal Science, Xinjiang Academy of Animal Sciences, Urumqi, Xinjiang China

**Keywords:** Chinese Merino (Xinjiang Type) sheep, Linkage disequilibrium, Effective population size, Genome-wide SNPs

## Abstract

Knowledge of linkage disequilibrium (LD) is important for effective genome-wide association studies and accurate genomic prediction. Chinese Merino (Xinjiang type) is well-known fine wool sheep breed. However, the extent of LD across the genome remains unexplored. In this study, we calculated autosomal LD based on genome-wide SNPs of 635 Chinese Merino (Xinjiang type) sheep by Illumina Ovine SNP50 BeadChip. A moderate level of LD (*r* ^2^ ≥ 0.25) across the whole genome was observed at short distances of 0–10 kb. Further, the ancestral effective population size (*N*_*e*_) was analyzed by extent of LD and found that *N*_*e*_ increased with the increase of generations and declined rapidly within the most recent 50 generations, which is consistent with the history of Chinese Merino sheep breeding, initiated in 1971. We also noted that even when the effective population size was estimated across different single chromosomes, *N*_*e*_ only ranged from 140.36 to 183.33 at five generations in the past, exhibiting a rapid decrease compared with that at ten generations in the past. These results indicated that the genetic diversity in Chinese Merino sheep recently decreased and proper protective measures should be taken to maintain the diversity. Our datasets provided essential genetic information to track molecular variations which potentially contribute to phenotypic variation in Chinese Merino sheep.

## Introduction

Linkage disequilibrium (LD) is the nonrandom co-occurrence of alleles within a chromosome or haplotype, i.e., statistical associations between alleles at separate loci that differ from the expectation for independent, and randomly sampled alleles (Wall and Pritchard 2003). The effective population size (*N*_*e*_) refers to the size of an idealized population that has the same dispersion of gene frequency under random genetic drift or the same degree of inbreeding as the population under consideration (Wright 1938). Both of these are crucial parameters for evaluations of population genetic diversity and can provide a powerful method to characterize and understand the genetic architecture underlying complex traits. With the advent of high-density SNP chips, high-throughput genotyping provides substantial data for more accurate estimate of LD and *N*_*e*_. These datasets help to improve our understanding of historical recombination within population (Reich and Lander 2001). LD over long distances can be used to evaluate recent *N*_*e*_, while LD at short distances can be applied to estimate ancient *N*_*e*_ (Hayes et al. 2003). Accordingly, *N*_*e*_ provides important information for the protection of populations.

In domestic livestock, many economic traits have been subjected to natural and artificial selection. These traits are passed on to offspring, and shape pattern of LD in populations. Thus, LD patterns throughout the genome reflect selection in individual breeds in domestic livestock species. In fact, the LD pattern in a population is the basis for genomic prediction, genome-wide association studies (GWAS), and quantitative trait loci (QTL) mapping for complex traits. The extent of LD has been examined in diverse taxa, including pigs (Amaral et al. 2008; Badke et al. 2012), horses (Corbin et al. 2010), cattle (Khatkar 2008; Mokry et al. 2014), and chickens (Qanbari et al. 2010). In the study of sheep, LD has been estimated using microsatellite markers across the genome in the early stage (Meadows et al. 2008).With the development of the Illumina Ovine SNP50 BeadChip, researchers investigated the extent of LD genome-wide in the wild big horn sheep (Miller et al. 2011), Dorper Spanish Churra sheep, Sunite (García-Gámez et al. 2012), German Mutton Merino, Dorper (Zhao et al. 2014), etc. Previous sheep studies revealed that LD in sheep persists for relatively shorter genomic distances compared with LD in other domestic species, and the behavior of LD varies among sheep breeds (Kijas et al. 2012). These findings provided insights into population structure, population diversity and genomic selection programs in sheep.

Chinese Merino (Xinjiang type) is the most well-known sheep breed for fine wool production in China. It has been breeding for highly specific purposes, such as fine and soft wool and hornless females, by the introduction of Australian Merino sheep via stages of grading, crossing, and collective reproduction. However, to date, the extent of LD across the genome in Chinese Merino sheep remains unexplored. The objective of this study was to investigate the behavior of LD in the genome of Chinese Merino (Xinjiang type) using ovine 50 K SNP panels.

## Materials and methods

### Animals

A total of 635 female Chinese Merino (Xinjiang type) sheep were randomly selected. All sheep were born between 2006 and 2012 in Bohu farm and Gongnaisi farm (Xinjiang, China). A very small sample of ear tissue was obtained for genomic DNA preparation using the saturated phenol–chloroform method (Sambrook and Russell 2002). DNA samples (2500 ng) with a 260/280 absorbance ratio of ≥1.8 and a DNA concentration of ≥50 ng/µl were submitted for genotyping. The DNA was genotyped using the Illumina Ovine SNP50 BeadChip (Illumina Inc., San Diego, CA,USA) (Teo et al. 2007), which contained 54,241 SNPs and at an average distance of 50.9 kb. The marker-QC (quality control) process included three steps: (i) control of the call rate (≥0.95); (ii) minor allele frequency (MAF) (≥0.05); (iii) correspondence with the Hardy–Weinberg equilibrium (HWE) (P-value > 0.00001).These parameters were calculated using PLINK v1.07.

### LD estimation and functional annotation of genes

Two measures, *r* ^2^ (William 1974) and *D*′ (Lewontin 1964), have been proposed to estimate the extent of LD. In this study, we used *r* ^2^ as a measure of LD because it is more robust to allele frequency variation than *D*′ (Ardlie et al. 2002) and population size has a greater influence on *D*′ than *r* ^2^ (Zhao et al. 2007). The *r* ^2^ value can be expressed as follows:

1 $${r^2} = \frac{{{{({{\text{P}}_{11}}{{\text{P}}_{12}} - {{\text{P}}_{12}}{{\text{P}}_{21}})}^2}}}{{{{\text{P}}_{{\text{A}}1}}{{\text{P}}_{{\text{A}}2}}{{\text{P}}_{{\text{B}}1}}{{\text{P}}_{{\text{B}}2}}}}$$

where P_A1_, P_A2_, P_B1_, and P_B2_ are the frequencies of each allele at loci A and B, and P_11_, P_12_, P_21_, and P_22_ are the frequencies of haplotypes A1B1, A1B2, A2B1, and A2B2, respectively.

PLINK (Purcell et al. 2007) includes a set of options to calculate pair-wise LD between SNPs and to process these data. The average *r* ^2^ values for distances of 0–25 kb, 25–50 kb, 50–100 kb, 100 − 500 kb, 0.5–1 Mb, 1–5 Mb, and 5–10 Mb in 635 Chinese Merino (Xinjiang type) sheep were calculated. LD was estimated as the mean *r* ^2^ value for each autosomal chromosome. Moreover, LD was calculated for randomly sampled populations (N = 30, 50, 100, 200, and 400). The four highest average *r* ^2^ values for a distance of 0–10 kb were detected on the four individual autosomes (OAR25, 24, 18, and 10). Genes were further evaluated when they were located between the pair of SNPs with *r* ^2^ ≥ 0.25 or were within 100 kb upstream or downstream of the pair of SNPs. The annotated gene lists were then used as inputs for a UniProt (http://www.uniprot.org/) analysis to obtain NCBI gene and protein IDs. Functional annotations of identified genes were made using BLAST2.0 and a parameter score of alpha greater than 0.6 was used to obtain gene ontology (GO) annotations (Conesa et al. 2005).

### Effective population size estimation and genetic diversity

LD data make it feasible to estimate *N*_*e*_. Sved (1971) described the relationship between LD and *N*_*e*_ using the following equation:

2 $$ {\text{E}}\left( {r^{2} } \right) = \frac{1}{{\left( {\upalpha + {\text{K}}N_{e} {\text{c}}} \right)}} + \frac{1}{{\text{n}}}$$

3 $$ {N_e} = \frac{{1{\text{ }}}}{{\left({r^2} - \frac{1}{{\text{n}}}\right){\text{kc}}}}{\text{ }} - {\text{ }}\frac{2}{{{\text{kc}}}}$$

Here, *r* ^2^ is the LD between different markers, *N*_*e*_ is the effective population size, and *c* is the genetic distance between various markers measured in Morgans; n is the chromosome experimental sample size; α = 1 in the absence of mutation and α = 2 if mutation is taken into account; k = 4 for autosomes and k = 2 for the X chromosome. In contemporary studies, physical distance is used instead of genetic distance to estimate population size. A physical distance of 100 kb is approximately equivalent to a genetic distance of 0.1 cM. The genetic distance reflects the effective population size at a certain number of generations in the past according to 1/2c (Hayes et al. 2003). Generally, the formula that assumes the absence of mutation is used to estimate *N*_*e*_. Hence, we used k = 4, and c = 1 to calculate *N*_*e*_.

It is generally agreed that abundant genetic diversity within a livestock species is a prerequisite for coping with potential changes in livestock farming conditions. Given that genetic diversity influences LD and ${N_{e}}$, Chinese Merino (Xinjiang type) were also used to analyze the proportion of polymorphic loci (*P*_*N*_), inbreeding coefficient and gene diversity (*H*_*E*_) using PLINK v1.07 (Purcell et al. 2007).

## Results

### SNP statistics

After quality control, we identified 46,062 SNPs in Chinese Merino (Xinjiang type) sheep distributed over 26 autosomal chromosomes. SNP information for each autosomal chromosome is summarized in Table 1. The total autosomal chromosome length was 2650.80 Mb, with an average chromosome length of 101.95 Mb; the longest *Ovis aries* autosomal chromosome was OAR1 (299.64 Mb) and the shortest was OAR24 (44.85 Mb). The average distance between adjacent SNPs was 57.49 kb; the longest adjacent SNP interval was 3.42 Mb within OAR10 and the shortest interval was observed in OAR14.

**Table 1:** Summary of SNPs included in the analysis

Chromosome	Length (Mb)	Number of SNP	Average SNP interval (Mb)	Longest SNP interval (Mb)	Shortest SNP interval (kb)
1	299.637	5180	0.058	0.913	5.291
2	263.109	4856	0.054	0.984	1.936
3	242.770	4409	0.055	1.358	5.326
4	127.202	2375	0.054	0.557	5.405
5	116.343	2089	0.056	0.843	0.065
6	129.054	2295	0.056	2.966	5.342
7	108.923	1952	0.056	0.105	5.303
8	97.814	1827	0.054	0.464	5.385
9	100.791	1884	0.053	0.406	5.406
10	94.128	1621	0.058	3.419	5.286
11	66.878	1048	0.064	0.611	5.300
12	86.402	1507	0.057	1.168	5.288
13	89.063	1492	0.060	0.903	5.478
14	69.303	1014	0.068	1.329	0.037
15	90.000	1481	0.061	1.855	5.414
16	77.051	1372	0.056	0.424	5.691
17	78.614	1254	0.063	0.563	5.413
18	72.434	1239	0.058	0.675	5.366
19	64.803	1104	0.059	0.473	5.678
20	55.394	983	0.056	1.069	5.499
21	55.476	781	0.071	2.422	5.475
22	55.747	960	0.058	2.258	5.312
23	66.685	998	0.067	0.715	5.385
24	44.851	648	0.069	0.344	5.514
25	48.288	885	0.055	0.590	5.514
26	50.044	808	0.062	1.692	0.915

### Extent of LD across the genome

Generally, at a distance of greater than 10 Mb, free recombination is assumed (De Roos et al. 2008; Zhao et al. 2014); accordingly, the range between markers was set at 0–10 Mb to estimate LD between SNP markers. The mean values of *r* ^*2*^ for genetic distances of 0–10 kb, 10–25 kb, 25–50 kb, 50–100 kb, 100–500 kb, 0.5–1 Mb, 1–5 Mb, and 5–10 Mb were 0.25, 0.17, 0.11, 0.07, 0.03, 0.02, 0.015, and 0.010, respectively, as summarized in Table 2. There is a decline in average values of *r* ^*2*^ with the increasing physical distance between SNPs.

**Table 2:** Statistical summary of linkage disequilibrium (LD) over various distances

Distance	Average r^2^	Number of SNP pairs
0–10 KB	0.25	1786
10–25 KB	0.17	6771
25–50 KB	0.11	19,821
50–100 KB	0.07	42,771
100–500 KB	0.03	330,617
0.5–1 MB	0.02	406,873
1–5 MB	0.015	2,398,635
5–10 MB	0.010	2,657,037

The extent of LD was quite different among individual chromosomes. The average *r* ^*2*^ for SNPs separated by intervals 0–10 kb, 10–25 kb, 25–50 kb, 50–100 kb, 100–500 kb, 0.5–1 Mb, 1–5 Mb and 5–10 Mb in each autosomal chromosome are presented in Table 3. However, when the genetic distance exceeded 100 kb, the mean *r* ^2^ values exhibited even smaller differences among chromosomes. The mean *r* ^2^ values were 0.017–0.022 within 0.5–1 Mb, and were maximal within 100 kb–1 Mb for OAR10.

**Table 3:** Statistical information for average *r* ^2^ as distance between pairs of SNP up to 10 Mb for the genome

CHR	SNP pairs distance							
	0–10 KB	10–25 KB	25–50 KB	50–100 KB	100–500 KB	0.5–1 MB	1–5 MB	5–10 MB
CHR1	0.247 ± 0.299	0.162 ± 0.219	0.105 ± 0.176	0.066 ± 0.122	0.028 ± 0.002	0.019 ± 0.029	0.016 ± 0.020	0.011 ± 0.013
CHR2	0.254 ± 0.306	0.183 ± 0.252	0.114 ± 0.186	0.075 ± 0.141	0.030 ± 0.058	0.020 ± 0.031	0.016 ± 0.021	0.011 ± 0.013
CHR3	0.238 ± 0.304	0.156 ± 0.220	0.114 ± 0.184	0.074 ± 0.133	0.032 ± 0.061	0.021 ± 0.033	0.016 ± 0.021	0.011 ± 0.013
CHR4	0.252 ± 0.326	0.169 ± 0.250	0.112 ± 0.181	0.073 ± 0.134	0.030 ± 0.056	0.019 ± 0.030	0.015 ± 0.019	0.010 ± 0.012
CHR5	0.253 ± 0.292	0.145 ± 0.224	0.098 ± 0.176	0.060 ± 0.113	0.030 ± 0.051	0.020 ± 0.031	0.016 ± 0.021	0.010 ± 0.012
CHR6	0.205 ± 0.267	0.144 ± 0.218	0.102 ± 0.156	0.062 ± 0.119	0.030 ± 0.055	0.020 ± 0.033	0.015 ± 0.020	0.011 ± 0.013
CHR7	0.239 ± 0.294	0.163 ± 0.236	0.108 ± 0.171	0.063 ± 0.116	0.028 ± 0.048	0.020 ± 0.030	0.016 ± 0.020	0.011 ± 0.012
CHR8	0.271 ± 0.326	0.150 ± 0.233	0.097 ± 0.152	0.058 ± 0.108	0.028 ± 0.048	0.019 ± 0.029	0.015 ± 0.019	0.010 ± 0.012
CHR9	0.255 ± 0.307	0.182 ± 0.244	0.104 ± 0.169	0.064 ± 0.119	0.028 ± 0.054	0.018 ± 0.026	0.015 ± 0.019	0.010 ± 0.012
CHR10	0.303 ± 0.346	0.181 ± 0.264	0.111 ± 0.196	0.073 ± 0.140	0.033 ± 0.074	0.022 ± 0.043	0.017 ± 0.025	0.012 ± 0.016
CHR11	0.201 ± 0.291	0.077 ± 0.157	0.072 ± 0.157	0.036 ± 0.092	0.029 ± 0.054	0.019 ± 0.030	0.014 ± 0.018	0.009 ± 0.010
CHR12	0.262 ± 0.277	0.170 ± 0.238	0.105 ± 0.180	0.062 ± 0.105	0.026 ± 0.049	0.018 ± 0.027	0.015 ± 0.018	0.010 ± 0.012
CHR13	0.237 ± 0.340	0.185 ± 0.250	0.117 ± 0.201	0.076 ± 0.141	0.031 ± 0.059	0.020 ± 0.030	0.015 ± 0.023	0.010 ± 0.012
CHR14	0.252 ± 0.312	0.102 ± 0.199	0.069 ± 0.147	0.037 ± 0.100	0.028 ± 0.053	0.019 ± 0.030	0.014 ± 0.019	0.009 ± 0.011
CHR15	0.210 ± 0.286	0.181 ± 0.260	0.104 ± 0.176	0.058 ± 0.110	0.026 ± 0.047	0.019 ± 0.028	0.015 ± 0.018	0.010 ± 0.012
CHR16	0.271 ± 0.341	0.154 ± 0.220	0.106 ± 0.172	0.061 ± 0.112	0.027 ± 0.045	0.020 ± 0.030	0.015 ± 0.019	0.010 ± 0.011
CHR17	0.209 ± 0.293	0.160 ± 0.244	0.086 ± 0.165	0.059 ± 0.123	0.028 ± 0.054	0.019 ± 0.030	0.015 ± 0.019	0.010 ± 0.012
CHR18	0.291 ± 0.309	0.144 ± 0.210	0.093 ± 0.165	0.072 ± 0.127	0.025 ± 0.043	0.017 ± 0.026	0.014 ± 0.018	0.010 ± 0.011
CHR19	0.183 ± 0.220	0.172 ± 0.240	0.123 ± 0.205	0.064 ± 0.122	0.028 ± 0.051	0.019 ± 0.029	0.014 ± 0.017	0.010 ± 0.011
CHR20	0.219 ± 0.298	0.142 ± 0.227	0.090 ± 0.145	0.052 ± 0.974	0.027 ± 0.045	0.018 ± 0.027	0.014 ± 0.018	0.010 ± 0.012
CHR21	0.248 ± 0.307	0.099 ± 0.191	0.060 ± 0.136	0.041 ± 0.094	0.025 ± 0.046	0.017 ± 0.025	0.014 ± 0.018	0.010 ± 0.011
CHR22	0.278 ± 0.325	0.164 ± 0.249	0.090 ± 0.161	0.059 ± 0.113	0.026 ± 0.049	0.017 ± 0.026	0.014 ± 0.017	0.009 ± 0.010
CHR23	0.203 ± 0.319	0.111 ± 0.175	0.092 ± 0.155	0.055 ± 0.105	0.025 ± 0.043	0.018 ± 0.027	0.014 ± 0.017	0.009 ± 0.011
CHR24	0.284 ± 0.292	0.091 ± 0.191	0.056 ± 0.133	0.033 ± 0.091	0.025 ± 0.041	0.0169 ± 0.025	0.013 ± 0.016	0.009 ± 0.010
CHR25	0.317 ± 0.352	0.142 ± 0.216	0.092 ± 0.153	0.057 ± 0.104	0.028 ± 0.054	0.018 ± 0.027	0.014 ± 0.017	0.009 ± 0.010
CHR26	0.154 ± 0.148	0.139 ± 0.207	0.099 ± 0.168	0.055 ± 0.105	0.028 ± 0.049	0.018 ± 0.028	0.014 ± 0.018	0.014 ± 0.011

CHR denotes chromosome

*r* ^2^ Means ± SE

### Sample size and LD estimates

**Fig. 1:**
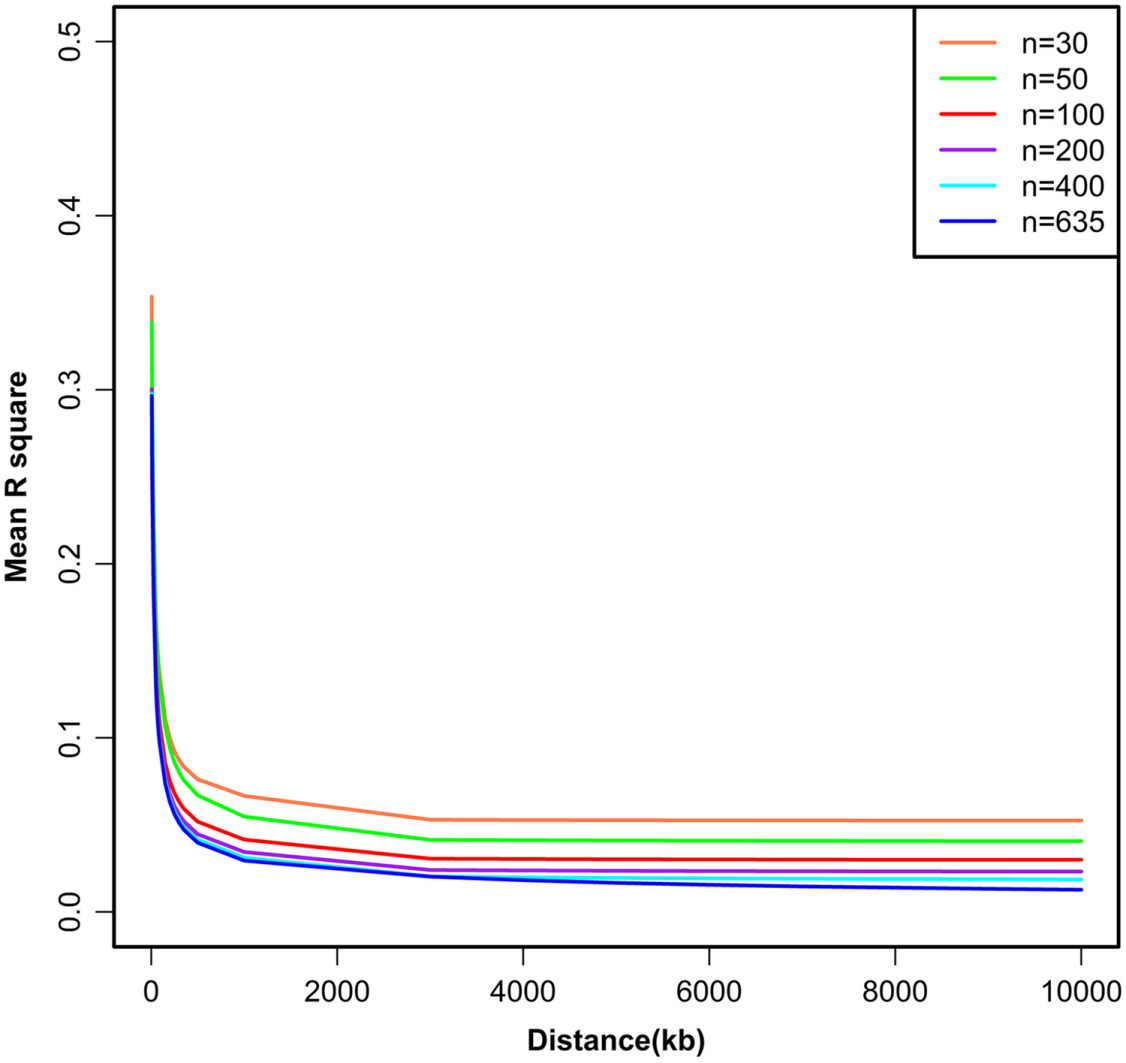
Linkage disequilibrium (LD) decay for six different sample sizes from Chinese Merino (Xinjiang type) sheep across all autosomal chromosomes. The *X* axis represents physical distance; the *Y* axis represents the average *r* ^2^

**Fig. 2:**
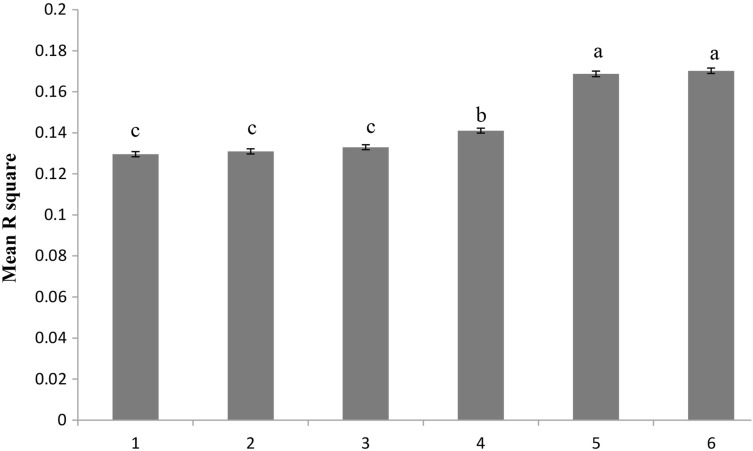
Average *r* ^2^ estimates at physical distance of 50-kb for six (N = 635, 400, 200, 100, 50, 30) population samples. Histograms 1, 2, 3, 4, 5, and 6 represent N = 635, 400, 200, 100, 50, and 30 on the *X* axis, respectively. The *Y* axis represents the average *r* ^2^. *Error bars* ± SE. *Bars* with the *same lower-case letter* were not significantly different, and those with *different lowercase letters* were significantly different. Statistical analysis was implemented in SPSS v19 using least-significant differences (LSD) and analysis of variance (ANOVA)

In order to determine the proper sample size for LD evaluation, population subsets of five different sizes (N = 30, 50, 100, 200, and 400) were randomly selected from the population and the level of LD was estimated for each sub-population. As shown in Fig. 1, as the sample size increased, the physical distance increased and the degree of LD tended to decrease. However, as shown in Fig. 2, within a physical distance of 50 kb, when N = 200 and physical distance was fixed at 50 kb, LD was not significantly different from what observed for the overall samples (P > 0.05).

### Functional annotation of genes

We performed a search for positional candidate genes for the regions with the four highest average *r* ^2^ values at 0–10 kb. In total, 280 Ensembl genes were found. Of these, 236 genes were located between pairs of SNPs (*r* ^2^ ≥ 0.25), and 44 genes were located within 100 kb upstream and downstream of pairs of SNPs. These genes were evaluated in a GO analysis. In total, 2353, 320, and 395 GO terms related to biological processes, cellular components, and molecular function, respectively, were identified. Ten GO terms (GO:0001942, GO:0043588, GO:0010631, GO: 0 002064, GO:0060429, GO:0043473,GO:0007173, GO:0016055, GO:0007219, and GO:0030509) were related to hair follicle development, skin development, epithelial cell migration, epithelial cell development, epithelium development, pigmentation, epidermal growth factor receptor signaling pathway, Wnt signaling pathway, Notch signaling pathway, and BMP signaling pathway, respectively (Table 4). Among them, 47 genes were related to epithelium and hair follicle development, of which 28 genes were located on OAR10, 14 genes were located on OAR18, 2 genes were located on OAR24, and 3 genes were located on OAR25.

**Table 4:** List of candidate genes in biological processes related to wool traits

Gene symbol	NCBI gene ID(chr)	GO term	GO name	Coordinates (bp)	Gene description
SETDB2	101116064 (10)	GO:0060429	Epithelium development	19,339,266–19,403,310	SET domain, bifurcated 2
VPS36	101121612 (10)	GO:0007219	Notch signaling pathway	21,876,927–21,896,255	Vacuolar protein sorting 36 homolog
FOXO1	101107877 (10)	GO:0060429 GO:0007173 GO:0016055	Epithelium development Epidermal growth factor receptor signaling pathway Wnt signaling pathway	22,237,525–22,238,862	Forkhead box O1
FREM2	101101900 (10)	GO:0060429	Epithelium development	23,560,959–23,715,430	FRAS1 related extracellular matrix protein 2
POSTN	101103329 (10)	GO:0007219	Notch signaling pathway	24,474,862–24,508,290	Periostin, osteoblast specific factor
SMAD9	101105084 (10)	GO:0060429 GO:0030509	Epithelium development BMP signaling pathway	25,005,441–25,028,218	SMAD family member 9
SPG20	101105830 (10)	GO:0030509	BMP signaling pathway	25,400,303–25,453,683	spastic paraplegia 20
KL	101110249 (10)	GO:0007173	Epidermal growth factor receptor signaling pathway	28,314,117–28,432,709	klotho
BRCA2	101109980 (10)	GO:0060429	Epithelium development	28,872,321–28,924,398	Breast cancer 2
FRY	101110521 (10)	GO:0060429 GO:0016055	Epithelium development Wnt signaling pathway	28,986,741–29,316,033	FRY microtubule binding protein
HMGB1	101112071 (10)	GO:0010631	Epithelial cell migration	30,583,831–30,588,285	High mobility group protein B1
POMP	101113603 (10)	GO:0060429	Epithelium development	31,695,500–31,707,769	Proteasome maturation protein
FLT1	443088 (10)	GO:0060429	Epithelium development	31,838,211–32,043,089	Fms-related tyrosine kinase 1
CDK8	101115551 (10)	GO:0060429 GO:0007219	Epithelium development Notch signaling pathway	33,310,224–33,390,657	Cyclin-dependent kinase 8
ATP8A2	101115632 (10)	GO:0043588	Skin development	33,449,390–33,534,534	ATPase phospholipid transporting 8A2
TNFRSF19	101116664 (10)	GO:0001942 GO:0043588 GO:0002064 GO:0060429	Hair follicle development Skin development Epithelial cell development Epithelium development	34,607,296–34,694,188	Tumor necrosis factor receptor superfamily, member 19
FGF9	101116844 (10)	GO:0007173 GO:0016055	Epidermal growth factor receptor signaling pathway Wnt signaling pathway	35,583,842–35,609,316	Fibroblast growth factor 9
LATS2	101117941 (10)	GO:0043588 GO:0060429 GO:0016055	Skin development Epithelium development Wnt signaling pathway	35,862,795–35,885,746	Large tumor suppressor kinase 2
IFT88	101118114 (10)	GO:0002064 GO:0060429 GO:0007219	Epithelial cell development Epithelium development Notch signaling pathway	36,048,434–36,103,776	Intraflagellar transport 88
KLF5	101123641 (10)	GO:0002064 GO:0060429	Epithelial cell development Epithelium development	48,247,059–48,264,092	Kruppel-like factor 5 (intestinal)
MYCBP2	101102993 (10)	GO:0043473	pigmentation	52,632,844–52,881,924	MYC binding protein 2, E3 ubiquitin protein ligase
SCEL	101103405 (10)	GO:0043588 GO:0060429	Skin development Epithelium development	53,073,650–53,289,357	Sciellin
EDNRB	443139 (10)	GO:0060429 GO:0043473	Epithelium development Pigmentation	53,508,345–53,534,498	Endothelin receptor type B
CCDC88C	101112005 (10)	GO:0060429 GO:0016055	Epithelium development Wnt signaling pathway	54,988,440–55,027,517	Coiled-coil domain containing 88C
EIF3F	101108947 (10)	GO:0007219	Notch signaling pathway	55,307,436–55,338,033	Eukaryotic translation initiation factor 3 subunit F
SPRY2	780494 (10)	GO:0060429 GO:0007173	Epithelium development Epidermal growth factor receptor signaling pathway	55,821,638–55,876,082	Sprouty RTK signaling antagonist 2
SLITRK6	101105831 (10)	GO:0002064 GO:0060429	Epithelial cell development Epithelium development	61,223,444–61,227,178	SLIT and NTRK like family member 6
EFNB2	101115469 (10)	GO:0010631 GO:0060429	Epithelial cell migration Epithelium development	81,604,311–81,649,792	Ephrin B2
FURIN	780454 (18)	GO:0007219	Notch signaling pathway	20,886,453–20,893,295	Furin (paired basic amino acid cleaving enzyme)
TJP1	443200 (18)	GO:0002064 GO:0060429	Epithelial cell development Epithelium development	27,453,888–27,538,010	Tight junction protein 1
SIN3A	101106112 (18)	GO:0010631 GO:0060429 GO:0016055	Epithelial cell migration Epithelium development Wnt signaling pathway	32,287,440–32,352,962	SIN3 transcription regulator family member A
CSK	101108956 (18)	GO:0060429 GO:0043473	Epithelium development Pigmentation	32,781,761–32,811,209	c-src tyrosine kinase
SET	101118680 (18	GO:0060429	Epithelium development	33,066,496–33,069,337	SET nuclear proto-oncogene
STRA6	101120527 (18)	GO:0060429	Epithelium development	33,348,745–33,395,596	Stimulated by retinoic acid 6
PML	101111580 (18)	GO:0060429	Epithelium development	33,464,431–33,515,176	Promyelocytic leukemia
TGIF1	101105544 (18)	GO:0060429	Epithelium development	34,892,531–34,894,053	TGFB-induced factor homeobox 1
PRKD1	101102016 (18)	GO:0010631	Epithelial cell migration	39,035,982–39,190,566	Protein kinase D1
HECTD1	100135435 (18)	GO:0060429	Epithelium development	40,626,204–40,697,112	HECT domain containing E3 ubiquitin protein ligase 1
SNX6	101118137 (18)	GO:0007173 GO:0016055	Epidermal growth factor receptor signaling pathway Wnt signaling pathway	43,896,580–43,993,579	Sorting nexin 6
PSMA6	100192424 (18)	GO:0007173 GO:0016055	Epidermal growth factor receptor signaling pathway Wnt signaling pathway	44,447,953–44,468,701	Proteasome subunit alpha 6
NFKBIA	780520 (18)	GO:0007219	Notch signaling pathway	44,535,115–44,538,036	NFKB inhibitor alpha
TGM7	101106629 (18)	GO:0043588 GO:0060429	Skin development Epithelium development	53,988,800–54,041,218	Transglutaminase 7
IFT140	101109504 (24)	GO:0060429	Epithelium development	1,173,624–1,189,733	Intraflagellar transport 140
LAT	101110725 (24)	GO:0010631 GO:0002064 GO:0007173 GO:0060429	Epithelial cell migration Epithelial cell development Epidermal growth factor receptor signaling pathway Epithelium development	25,748,047–25,870,792	Linker for activation of T-cells
RASGEF1A	101120047 (25)	GO:0007173	Epidermal growth factor receptor signaling pathway	13,514,772–13,521,910	RasGEF domain family member 1 A
CDH23	101107570 (25)	GO:0002064 GO:0060429	Epithelial cell development Epithelium development	27,373,089–27,820,109	Cadherin-related 23
DLG5	101110731 (25)	GO:0010631 GO:0002064 GO:0060429	Epithelial cell migration; Epithelial cell development Epithelium development	33,385,018–33,473,757	Discs, large homolog 5 (Drosophila)

### Effective population size and genetic diversity

**Fig. 3:**
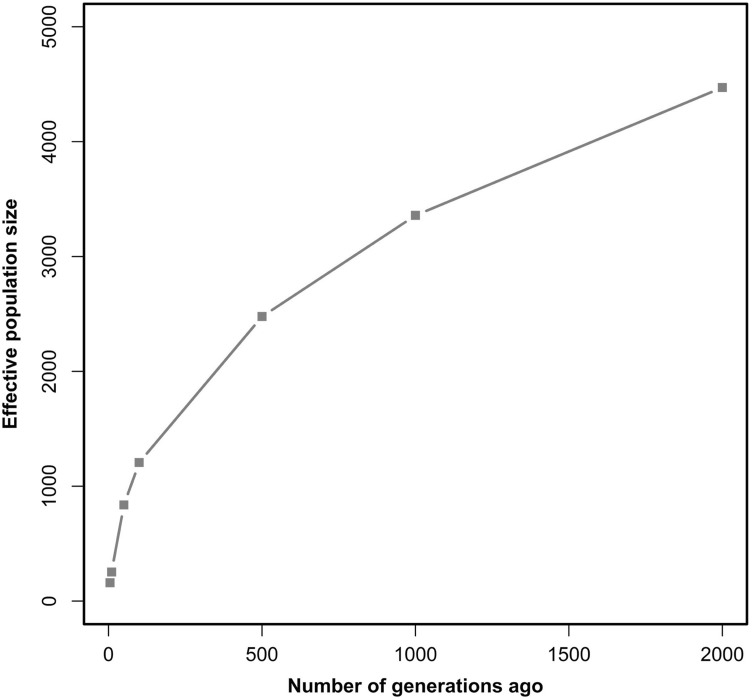
Estimated *N*_*e*_ for Chinese Merino (Xinjiang type) sheep over time based on linkage disequilibrium data. *Y* axis represents the effective population size; *X* axis represents time elapsed (number of generations)

**Fig. 4:**
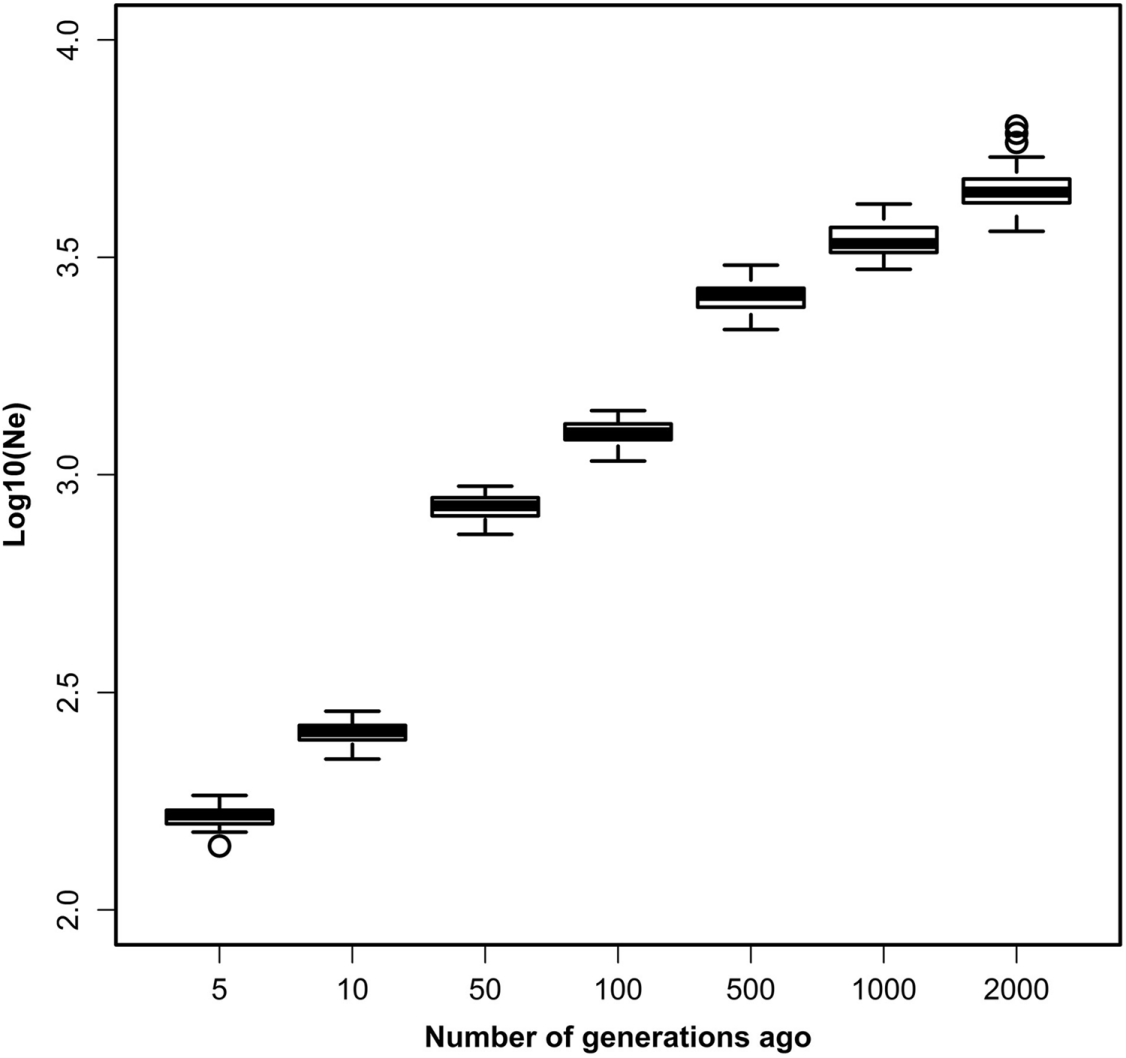
Boxplot represents the trend in log10(*N*_*e*_) over time. The variability at each time point reflects variation in the estimates among the 26 autosomes. The *X* axis represents the number of generations in the past; the *Y* axis represents log10(*N*_*e*_)

We calculated *N*_*e*_ of seven generations of whole autosomal and individual autosomes, respectively. Table 5 summarizes the *N*_*e*_ of Chinese Merino (Xinjiang type) over time. Estimates of *N*_*e*_ for Chinese Merino (Xinjiang type) showed an increasing trend with the increase of generations, as shown in Fig. 3. *N*_*e*_ at 2000 generations ago was approximately 4,471.67 and decreased to 159.77 at five generations ago. The effective population size of Chinese Merino (Xinjiang type) differed depending on chromosome, and this was mainly caused by differences among chromosomes in LD between SNP markers (Table 6; Fig. 4). In the 5th generation (from the present), *N*_*e*_ was 140.36–183.33. This indicated that the effective population size of Chinese Merino (Xinjiang type) decreased over time, and this decrease was more rapid at 100–50 years ago and recently stabilized. Within the same generation, there were significant differences in effective population size between chromosomes (ANOVA, P < 0.001, *F-*statistics, SPSSv19). When we calculated the effective population size for each chromosome, we found the value of generations became bigger. Within the current generation, OAR10 exhibited the smallest effective population compared with the other chromosomes. Due to OAR3 exhibit small effective population after 50 generations, OAR3 genetic information was selected in Chinese Merino (Xinjiang type) since 50 generations.

**Table 5:** Statistical summary of the effective population sizes of sheep

	SNP pairs distance						
	25 KB	50 KB	100 KB	500 KB	1 MB	5 MB	10 MB
Genetic distance	0.00025	0.0005	0.001	0.005	0.01	0.05	0.1
Generations ago	2000	1000	500	100	50	10	5
N_e_	4471.67	3358.00	2477.67	1206.44	837.07	253.51	159.77

Genetic distance: Morgan

*N*_*e*_ effective population size

**Table 6:** Effective population size of Chinese Merino (Xinjiang type) sheep over time estimated by different autosomal chromosomes

CHR	Generations						
	2000	1000	500	100	50	10	5
1	4473.99	3302.02	2469.60	1225.03	831.14	246.26	157.76
2	4034.74	3096.04	2221.71	1128.94	781.77	237.21	152.78
3	4782.04	3300.12	2331.18	1106.14	753.72	232.59	151.07
4	4257.22	3204.97	2311.83	1148.25	799.50	247.53	161.54
5	4219.43	3245.12	2428.83	1187.06	803.48	241.31	155.83
6	5380.44	3705.36	2699.85	1204.32	804.64	245.25	157.86
7	4105.49	3221.11	2496.41	1220.42	825.24	246.26	157.76
8	4308.70	3537.53	2658.67	1256.06	846.30	252.73	161.65
9	4145.04	3244.30	2496.15	1240.66	866.59	257.47	165.29
10	3858.85	3134.65	2281.99	1075.61	729.60	222.27	140.36
11	5812.27	3531.54	2623.76	1230.01	833.19	260.53	170.43
12	4294.37	3278.60	2549.43	1288.44	885.75	259.55	165.40
13	3929.02	3035.82	2157.23	1081.27	761.41	245.00	158.34
14	4790.39	3482.79	2543.48	1221.29	835.29	256.60	168.31
15	4326.74	3324.72	2645.53	1308.70	878.24	260.25	166.21
16	4709.33	3492.97	2632.91	1287.08	855.59	254.07	163.91
17	4957.46	3770.99	2650.23	1262.34	851.89	256.57	164.39
18	4460.37	3701.68	2517.35	1301.85	896.69	265.78	169.56
19	4754.47	2968.20	2370.55	1204.71	832.40	261.01	167.05
20	4877.87	3948.13	3035.15	1367.48	917.02	268.13	167.80
21	4620.63	3712.66	2734.12	1354.49	925.61	271.74	172.57
22	4267.18	3552.59	2685.41	1327.79	913.80	275.73	177.36
23	6339.45	4193.07	2918.57	1404.77	941.00	275.63	176.53
24	3630.12	3282.15	2648.25	1366.43	936.47	286.40	183.33
25	4721.48	3788.17	2862.57	1304.14	882.97	274.49	176.85
26	6105.10	4064.54	2988.76	1320.36	885.42	263.82	167.80

In general, sheep breeds have roughly comparable levels of polymorphism, with a polymorphic loci (*P*_*N*_) value ranging from 0.767 (Border Leicester) (Kijas et al. 2014) to 0.973 (Badger Faced) (Beynon et al. 2015). Accordingly, expected heterozygosity (*H*_*E*_) was relatively high in all sheep breeds (range 0.306–0.380) (Beynon et al. 2015). For the 46,062 SNPs analyzed in this study, estimates of polymorphic loci and expected heterozygosity were 0.994 and 0.393, respectively. The mean inbreeding coefficient was 0.017, which was lower than that of other sheep breeds in previous studies (Kijas et al. 2012).

## Discussion

LD maps have improved the power and precision of association mapping, and are used to define optimal marker spacing. In this study, we analyzed LD in 635 Chinese Merino (Xinjiang type) using 46,062 SNPs distributed over 26 autosomal chromosomes. LD was distinct from populations, and with the sample size increased, the extent of LD droped down. When N = 200, the estimated LD within a physical distance of 50 kb was close to that of the general population.

We used *r* ^2^ as a measure of LD because it is more robust to allele frequency variation than *D*′ (Ardlie et al. 2002). For Chinese Merino (Xinjiang type) sheep, a moderate level of LD (*r* ^2^ ≥ 0.25) was only observed at marker distances of 0–10 kb. When moving from 10 to 500 kb, average *r* ^2^ declined from 0.25 to 0.03. Marker pairs with *r* ^2^ ≥ 0.25 were, on average, separated by 10 kb. However, not all marker pairs within 10 kb featured by *r* ^2^ ≥ 0.25. Our calculated *r* ^2^ values were smaller than those observed in Poll Dorset and Border Leicester sheep, but greater than those of Australia Merino and Suffolk breeds for 0–10 kb (Kijas et al. 2014). Compared to LD calculated by Zhao et al. (2014) within 0–10 Mb, Chinese Merino (Xinjiang type) LD was less than that of German Mutton Merino, but greater than that of Sunite sheep. Meanwhile, the extent of LD was different from the same fragment length on different autosomal chromosomes, which was consistent with previous results of sheep and cows (Edea et al. 2015; Lu et al. 2012; Zhao et al. 2014). Potential causes of these differences include variations in recombination rates between and within chromosomes, heterozygosity, genetic drift, and selection for economic traits.

Meat, wool, and milk are the most important economic traits in livestock production. In our analysis, we found 280 genes in 0–10 kb with maximum *r* ^2^ values for the first four autosomes (OAR25, 24, 18, and 10), of which 47 genes were involved in biological processes related to wool traits, particularly *TNFRSF19, FOXO1, SMAD9*, and *FGF9. TNFRSF19* is located on OAR10 from 34,589,405 to 34,690,170 bp in sheep. It is a member of the tumor necrosis factor receptor superfamily (Hu et al. 1999) and plays a crucial role in hair follicle development, skin development, epithelial cell development, and epithelium development (Kojima et al. 2000; Pispa et al. 2008). *FOXO1* functions on epithelium development and the epidermal growth factor receptor signaling pathway, as well as Wnt signaling pathway (Guan et al. 2015; Mori et al. 2014; Xu et al. 2015). This gene, on OAR10 from 22,235,779 to 22,242,617 bp in sheep, belongs to the forkhead transcription factors. The *SMAD9* gene, located on OAR10 from 25,001,877 to 25,031,583 bp in sheep, belongs to the SMAD family. It is involved in epithelium development and the BMP signaling pathway (Tsukamoto et al. 2014; Yoshimoto et al. 2005). *FGF9* located on OAR10 from 35,583,772 to 35,610,032 bp, is a member of the fibroblast growth factor family. This gene is involved in the epidermal growth factor receptor signaling pathway and the Wnt signaling pathway (Loke et al. 2013; Zheng et al. 2012). Wang et al. (2014) found 28 significant SNPs in Chinese Merino (Junken type) mainly through a GWAS approach and from these SNPs, 25 genes were found to be affected. The 25 genes are dispersed located on OAR1, OAR2, OAR3, OAR4, OAR5, OAR6, OAR7, OAR8, OAR9, OAR10, OAR13, OAR23 and OAR25. In contrast, we found 280 genes locating on four chromosomes, and these genes showed a non-random distribution. The main reason is that Wang et al. used quantitative trait correlation analysis to detect genes under selection, while we detected genes directly from the perspective of genetic linkage. The genetic difference of these two breeds may also contribute to the distinction of the results. Wang et al. found candidate genes harboured in OAR10 and OAR25. Noteablly, after careful study, we also found some of these candidate genes located at LD blocks, such as CFDP2, TPTE2, NBEA as well as SLC25A5.

The LD pattern in a population is generally shaped by selection, mutation rate, recombination rate, consanguinity, genetic drift, and other factors. Moreover, LD and effective population size are closely related to each other. These data can help us understand the evolutionary history and genetic mechanisms of complex traits formation (Reich and Lander 2001). In the present study, we found that level of LD in Chinese merino sheep was lower and the effective population size of Chinese Merino (Xinjiang type) increased with the number of generations increase. The effective population size within the most recent 100 generations decreased more slowly, and a relatively rapid decrease was observed after 100 generations. The rate of decay increased at 50 generations, which corresponds to the time of Chinese Merino (Xinjiang type) breeding. *N*_*e*_ estimated using each chromosome increased with the number of generations raised and decreased with LD drop down. These results were consistent with the analysis of *N*_*e*_ for the whole autosomal genome. The effective population size estimated by OAR10 was smaller than other autosomes. The corresponding *r* ^2^ value was high. QTL related to horn characters is located on this chromosome, and as expectation, we detected *RXFP2* gene, which is a candidate for horn phenotypes (Montgomery et al. 1996). This was consistent with the history of long-term breeding of hornless Merino sheep. OAR3 exhibited a relatively small effective population size after 50 generations (corresponding to the completion of Xinjiang type Merino breeding). This chromosome has QTL related to greasy fleece weight and staple length (Ponz et al. 2001). Keratin family genes related to wool quality were also located on this chromosome (McLaren et al. 1997). Factors that influence LD also influence *N*_*e*_ according to the formulae used to estimate these parameters. For example, artificial selection will lead to higher LD, and a high LD would result in decreased *N*_*e*_. Therefore, the small effective population estimated for OAR10 and three may be explained by selection for characters related to wool quality on the basis of hornless sheep. This breed had a larger effective population size than that of Sunite sheep calculated by Zhao et al. (2014) within the same 2000 generations. This indicated that Chinese Merino (Xinjiang type) sheep may have originated from a large effective population. These results may be related to natural selection of Chinese Merino sheep. We did not estimate present-day *N*_*e*_ in this study. Therefore, we selected 10 Mb as the maximal fragment length. The average *r* ^2^ for Chinese Merino (Xinjiang type) population was 0.010. *N*_*e*_ of Chinese Merino (Xinjiang type) was determined in this estimate, which may estimates the effective population size in current populations.

For genetic diversity, Al-Mamun et al. (2015) examined crosses of Merino × Border Leicester with Poll Dorset (MxBxP; N = 231) and reported an expected heterozygosity of 0.38, but an estimated *N*_*e*_ of 152, which is lower than that of other sheep breeds. In our study, Chinese Merino (Xinjiang type) displayed the highest genetic diversity as measured by polymorphic loci and gene diversity. Historically, Chinese Merino (Xinjiang type) did not undergo intensive selection, unlike the other sheep breeds. Hence, it is reasonable to assume that Chinese Merino (Xinjiang type) sustain higher levels of genetic variability compare with other sheep breeds. However, the *N*_*e*_ of Chinese Merino (Xinjiang type) was much lower than that of other sheep breeds, particularly Badger Faced and Llandovery White Faced sheep breeds (Beynon et al. 2015). This difference may be explained by the history of Chinese Merino sheep breeding, in which a few Australia Merino rams were introduced, led to a decrease in *N*_*e*_, while the large number of native ewes facilitated the maintenance of high genetic diversity in Chinese Merino.

In short, in Chinese Merino (Xinjiang type) population, LD decayed as genomic distance increased and the observed LD values were consistent with previously estimates in other sheep populations. After 50 generations (in the past), *N*_*e*_ decreased more rapidly than in recent generations and proper protective measures should be taken to maintain the breed resources genetic diversity.

## References

[CR1] Al-Mamun HA, Clark SA, Kwan P, Gondro C (2015). Genome-wide linkage disequilibrium and genetic diversity in five populations of Australian domestic sheep. Genet Sel Evol.

[CR2] Amaral AJ, Megens HJ, Crooijmans RP, Heuven HC, Groenen MA (2008). Linkage disequilibrium decay and haplotype block structure in the pig. Genetics.

[CR3] Ardlie KG, Kruglyak L, Seielstad M (2002). Patterns of linkage disequilibrium in the human genome. Nat Rev Genet.

[CR4] Badke YM, Bates RO, Ernst CW, Schwab C, Steibel JP (2012). Estimation of linkage disequilibrium in four US pig breeds. BMC Genom.

[CR5] Beynon SE, Slavov GT, Farre MS, Waddams K, Davies B, Haresign W, Kijas J, MacLeod IM, Newbold CJ, Davies L, Larkin DM (2015). Population structure and history of the Welsh sheep breeds determined by whole genome genotyping. BMC Genet.

[CR6] Conesa A, Gotz S, Garcia-Gomez JM, Terol J, Talon M, Robles M (2005). Blast2GO: a universal tool for annotation, visualization and analysis in functional genomics research. Bioinformatics.

[CR7] Corbin LJ, Blott SC, Swinburne JE, Vaudin M, Bishop SC, Woolliams JA (2010). Linkage disequilibrium and historical effective population size in the Thoroughbred horse. Anim Genet.

[CR8] De Roos AP, Hayes BJ, Spelman RJ, Goddard ME (2008). Linkage disequilibrium and persistence of phase in Holstein-Friesian, Jersey and Angus cattle. Genetics.

[CR9] Edea Z, Dadi H, Dessie T, Lee S-H, Kim K-S (2015). Genome-wide linkage disequilibrium analysis of indigenous cattle breeds of Ethiopia and Korea using different SNP genotyping BeadChips. Genes Genom.

[CR10] García-Gámez E, Sahana G, Gutiérrez-Gil B, Arranz JJ (2012). Linkage disequilibrium and inbreeding estimation in Spanish Churra sheep. BMC Genet.

[CR11] Guan H, Tan P, Xie L, Mi B, Fang Z, Li JYJ, Liao H, Li F (2015). FOXO1 inhibits osteosarcoma oncogenesis via Wnt/beta-catenin pathway suppression. Oncogenesis.

[CR12] Hayes B, Visscher P, McPartlan H, Goddard M (2003). Novel multilocus measure of Linkage Disequilibrium to estimate past effective population size. Genome Res.

[CR13] Hu SM, Tamada K, Ni J, Vincenz C, Chen L (1999). Characterization of TNFRSF19, a novel member of the tumor necrosis factor receptor superfamily. Genomics.

[CR14] Khatkar MS, Nicholas F, Collins AR, Zenger KR, Cavanagh JA, Barris W, Schnabel RD, Taylor JF, Raadsma HW (2008). Extent of genome-wide linkage disequilibrium in Australian Holstein-Friesian cattle based on a high-density SNP panel. BMC Genom.

[CR15] Kijas JW, Lenstra JA, Hayes B, Boitard S, Neto LRP, San Cristobal M, Servin B, McCulloch R, Whan V, Gietzen K, Paiva S, Barendse W, Ciani E, Raadsma H, McEwan J, Dalrymple B (2012). Genome-wide analysis of the world’s sheep breeds reveals high levels of historic mixture and strong recent selection. PLoS Biol.

[CR16] Kijas J, Porto-Neto L, Dominik S, Reverter A, Bunch R, McCulloch RHB, Brauning R, McEwan J (2014). Linkage disequilibrium over short physical distances measured in sheep using a high-density SNP chip. Anim Genet.

[CR17] Kojima T, Morikawa Y, Copeland NG, Gilbert DJ, Jenkins NA, Senba E, Kitamura T (2000). TROY, a newly identified member of the tumor necrosis factor receptor superfamily, exhibits a homology with Edar and is expressed in embryonic skin and hair follicles. J Biol Chem.

[CR18] Lewontin RC (1964). The interaction of selection and linkage. I. general considerations; heterotic models. Genetics.

[CR19] Loke J, Pearlman A, Radi O, Zuffardi O, Giussani U, Pallotta R, Camerino G, Ostrer H (2013). Mutations in MAP3K1 tilt the balance from SOX9/FGF9 to WNT/ -catenin signaling. Hum Mol Genet.

[CR20] Lu D, Sargolzaei M, Kelly M, Li C, Vander Voort G, Wang Z, Plastow G, Moore S, Miller SP (2012). Linkage disequilibrium in Angus, Charolais, and Crossbred beef cattle. Front Genet.

[CR21] McLaren R, Rogers G, Davies K, Maddox J, Montgomery G (1997). Linkage mapping of wool keratin and keratin-associated protein genes in sheep. Mamm Genome.

[CR22] Meadows JR, Chan EK, Kijas JW (2008). Linkage disequilibrium compared between five populations of domestic sheep. BMC Genet.

[CR23] Miller JM, Poissant J, Kijas JW, Coltman DW (2011). A genome-wide set of SNPs detects population substructure and long range linkage disequilibrium in wild sheep. Mol Ecol Resour.

[CR24] Mokry F, Buzanskas M, Mudadu M, Grossi D, Higa R, Ventura RV, de Lima A, Sargolzaei M (2014). Linkage disequilibrium and haplotype block structure in a composite beef cattle breed. BMC Genom.

[CR25] Montgomery GW, Henry HM, Dodds KG, Beattie AE, Wuliji T, Crawford AM (1996). Mapping the Horns (Ho) locus in sheep a further locus controlling horn development in domestic. J Hered.

[CR26] Mori R, Tanaka K, de Kerckhove M, Okamoto M, Kashiyama K, Kim S, Kawata T, Komatsu T, Park S, Ikematsu K, Hirano A, Martin P, Shimokawa I (2014). Reduced FOXO1 expression accelerates skin wound healing and attenuates scarring. Am J Pathol.

[CR27] Pispa J, Pummila M, Barker PA, Thesleff I, Mikkola ML (2008). Edar and Troy signalling pathways act redundantly to regulate initiation of hair follicle development. Hum Mol Genet.

[CR28] Ponz R, Moreno C, Allain D, Elsen JM, Lantier F, Lantier I, Brunel J, Perez-Enciso M (2001). Assessment of genetic variation explained by markers for wool traits in sheep via a segment mapping approach. Mamm Genome.

[CR29] Purcell SNB, Todd-Brown KTL, Ferreira M, Bender DA, Maller J, Sklar P, de Bakker PI, Daly MJ, Sham PC (2007). PLINK: a tool set for whole-genome association and population-based linkage analyses. Am J Hum Genet.

[CR30] Qanbari S, Hansen M, Weigend S, Preisinger R, Simianer H (2010). Linkage disequilibrium reveals different demographic history in egg laying chickens. BMC Genet.

[CR31] Reich DE, Lander ES (2001). On the allelic spectrum of human disease. Trends Genet.

[CR32] Sambrook J, Russell DW (2002). Molecular cloning: a laboratory manual.

[CR33] Sved J (1971). Linkage disequilibrium and homozygosity of chromosome segments in finite populations. Theor Popul Biol.

[CR34] Teo YY, Inouye M, Small KS, Gwilliam R, Deloukas P, Kwiatkowski DP, Clark TG (2007). A genotype calling algorithm for the Illumina BeadArray platform. Bioinformatics.

[CR35] Tsukamoto S, Mizuta T, Fujimoto M, Ohte S, Osawa K, Miyamoto A, Yoneyama K, Murata E, Machiya A, Jimi E, Kokabu S, Katagiri T (2014). Smad9 is a new type of transcriptional regulator in bone morphogenetic protein signaling. Sci Rep.

[CR36] Wall JD, Pritchard JK (2003). Haplotype blocks and linkage disequilibrium in the human genome. Nat Rev Genet.

[CR37] Wang ZP, Zhang H, Yang H, Wang SZ, Rong EG, Pei W, Li H, Wang N (2014). Genome-wide association study for wool production traits in a chinese merino sheep population. PLoS ONE.

[CR38] William GH (1974). Estimation of linkage disequilibrium in randomly mating populations. Heredity.

[CR39] Wright S (1938). Size of population and breeding structure in relation to evolution. Science.

[CR40] Xu ZH, Shun WW, Hang JB, Gao BL, Hu JA (2015). Posttranslational modifications of FOXO1 regulate epidermal growth factor receptor tyrosine kinase inhibitor resistance for non-small cell lung cancer cells. Tumour Biol.

[CR41] Yoshimoto A, Saigou Y, Higashi Y, Kondoh H (2005). Regulation of ocular lens development by Smad-interacting protein 1 involving Foxe3 activation. Development.

[CR42] Zhao H, Nettleton D, Dekkers JC (2007). Evaluation of linkage disequilibrium measures between multi-allelic markers as predictors of linkage disequilibrium between single nucleotide polymorphisms. Genet Res.

[CR43] Zhao FP, Wang GK, Zeng T, Wei CH, Zhang L, Wang HH, Zhang SZ, Liu RZ, Liu Z, Du LX (2014). Estimations of genomic linkage disequilibrium and effective population sizes in three sheep populations. Livest Sci.

[CR44] Zheng Z, Kim J, Choi MJ, Goo B, Chun SI, Cho SB (2012). Histometric changes and epidermal FGF9 expression in carbon photoenhancer-assisted Nd:YAG laser treatment. J Dermatolog Treat.

